# The Avian Collection of the Zoological Museum of the University of Athens (ZMUA)

**DOI:** 10.3897/BDJ.4.e10598

**Published:** 2016-11-01

**Authors:** Gabriella Papastefanou, Anastasios Legakis, Igor Shogolev

**Affiliations:** ‡ Hellenic Centre for Marine Research, Heraklion, Crete, Greece; § Zoological Museum of the University of Athens, Athens, Greece

**Keywords:** Aves, zoological collection, occurrence data, LifeWatchGreece

## Abstract

**Background:**

The Zoological Museum of the University of Athens (ZMUA) was established in 1858. It is the oldest natural history museum of Greece. The museum began its operation with the acquisition of a core collection and has been expanding ever since. One of the most substantial parts of the museum's collection consists of the avian exhibits, originating from around the world.

**New information:**

Today, the avian collection consists of 2,948 specimens, preserved mostly through taxidermy, along with a significant number of eggs. The birds have been collected from around the world. A substantial part of the collection consists of individuals originating from Greece, Brazil, Canada and Australia. Having this valuable source of biogeographic information and a potential reserve of historical genetic diversity, ZMUA presents here the contents of the avian collection.

## Introduction

The Zoological Museum of the University of Athens (ZMUA) was established in 1858 and is the oldest museum of its kind in Greece. Starting from a core collection donated to the museum by the Physiographic Society that already existed since 1836 (Legakis 1983), it has been continuously expanding its collections ever since. Today, the Museum is administered by the Department of Biology of the University of Athens.

One of the most substantial parts of the museum's collection consists of the avian exhibits, originating from around the world. The oldest specimen dates back to 1806 while new inputs are purchased or donated up until today. The collection is mainly constituted by specimens collected primarily by the staff of the museum, donations or by fortuitous findings. The complete number of the birds in the collection today reaches 2,948 specimens preserved mainly through taxidermy or freezing. Avian skeletons are not included in this list.

**Figure 1.**
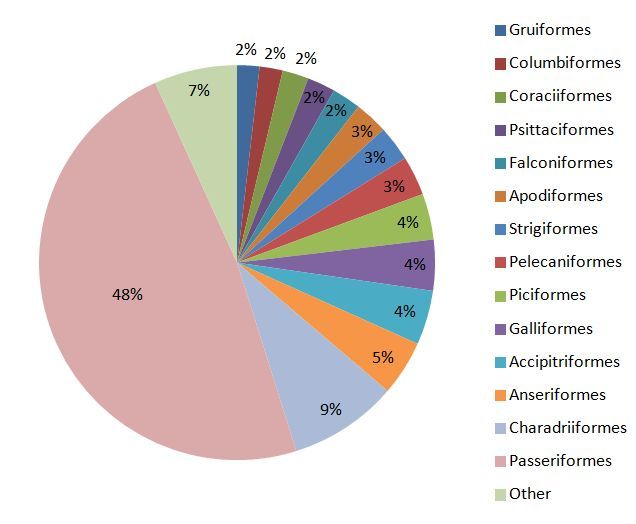
Distribution of the avian specimens represented in the ZMUA collection to taxonomic orders.

The vast majority of the avian specimens in the ZMUA collection belong to Passeriformes (48%) followed by Charadriiformes (8.9%), Anseriformes (4.5%), Accipitriformes (4.4%) and Galliformes (4.1%) (Fig. 1). Passeriformes are represented by 83 families, with Emberizidae and Thraupidae counting most representatives. There are no type specimens or extinct species in the avian depository of the museum. Thirty four (34) of the species available in the collection are marked as Vulnerable by IUCN Red List of Threatened Species, 3 of them as Endangered and 5 species as Critically Endangered. The list of these species is presented in Table 1. This valuable source of preserved phenotypes, potential genetic diversity and biogeographic data can be of great contribution where conservation practices are concerned. Therefore, ZMUA publishes the metadata and data of the contents of the avian collection, in the context of the LifewatchGreece Research Infrastructure. A complete species list is provided in the supplementary material (Suppl. material 1).

ZMUA also possess a significant number of avian eggs that have not been studied yet. This collection contains eggs from a wide taxonomic range and different localities in Greece and abroad, as well as a small number of bird nests.

## Sampling methods

### Study extent

ZMUA is a museum hosting specimens from around the world. Particularly, the avian collection contains 2,948 exhibits from 214 different localities.

### Sampling description

Specimens deposited in the ZMUA collection are the result of collections made by the staff of the museum, donations and fortuitous findings. Therefore, no specific sampling protocol has been followed. Ιf in a certain sampling expedition a protocol was followed then this information is not available. Specimens are basically preserved through taxidermy. Newer deposits are stored in frozen condition.

### Quality control

Before deposition to the museum collection, associated information of the specimens has being sought. However, owing to frequent relocations of the Zoological Museum of the University of Athens and several misfortunate events, some of the exhibits and the relevant meta-data have been lost. Digitization, quality control checks and data analysis were made possible in the context of LifeWatchGreece project. All preserved information has been curated according to Chapman (2005). Scientific names have been updated following the Integrated Taxonomic Information System (ITIS) taxonomy. None of the data connected to the specimens contained coordinates. Therefore, all locations were manually georeferenced, based on the locality names, description and dates, following georeferencing guidelines by Chapman and Wieczorek (2006).

## Geographic coverage

### Description

**Figure 2.**
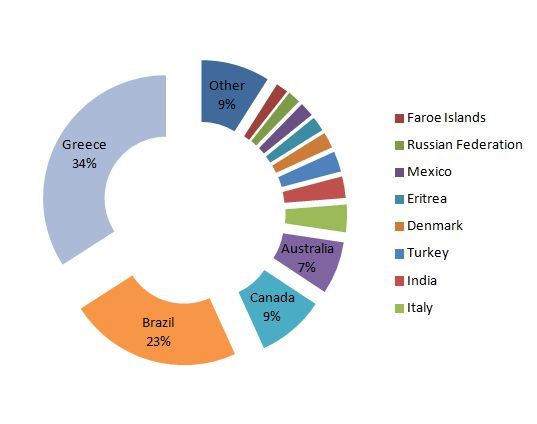
Distribution of the geographic origin percentage of avian specimens deposited in ZMUA collection. Parts without labels on the chart represents percentages below 5%. Countries pooled in the “other” category refer to localities with representatives’ percentage below 1%.

The avian specimens deposited in the ZMUA collection have worldwide origin. Particularly, the exhibits’ origin is from 214 different localities that belong to 52 countries in all continents. Most of them were collected from Greece (34%), while the second largest collection comes from Brazil (23%). Furthermore, a substantial part of the collection comes from Canada (9%) and Australia (7%) (Fig. 2). Specimens originating from Greece were collected from 53 different localities. Most of the birds come from Attica (79%) where the Museum is situated, followed by Mount Taygetos in Peloponnese (5%) and Mount Olympus in Macedonia (3%).

## Taxonomic coverage

### Taxa included

**Table:** 

Rank	Scientific Name	Common Name
class	Aves	Birds
order	Accipitriformes﻿	
order	Apodiformes	
order	Anseriformes	
order	Bucerotiformes	
order	Charadriiformes	
order	Ciconiiformes﻿	
order	Columbiformes	
order	Coraciiformes	
order	Craciformes	
order	Cuculiformes	
order	Coliiformes	
order	Falconiformes	
order	Galbuliformes	
order	Galliformes	
order	Gaviiformes	
order	Gruiformes	
order	Musophagiformes	
order	Passeriformes	
order	Pelecaniformes	
order	Phoenicopteriformes	
order	Piciformes	
order	Podicipediformes	
order	Procellariiformes	
order	Psittaciformes	
order	Pteroclidiformes	
order	Sphenisciformes	
order	Strigiformes	
order	Struthioniformes	
order	Suliformes	
order	Tinamiformes	
order	Trogoniformes	
order	Upupiformes	

## Temporal coverage

**Data range:** 1806 1 01 – 2010 1 26.

### Notes

**Figure 3.**
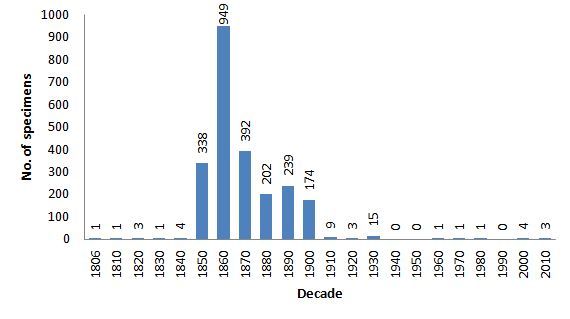
Temporal distribution of the specimen records in the ZMUA collection.

The oldest specimen was collected in 1806, while the collection is still being enriched. Most of the exhibits for which the information is available, were collected during the 19^th^ century (Fig. 3). 40% of these date back to the 1860s (Krüper 1862).

## Collection data

### Collection name

Avian collection of the Zoological Museum of the University of Athens (ZMUA)

### Collection identifier

Zoological Museum of the University of Athens (ZMUA)

### Specimen preservation method

Mounting, Freezing

## Usage rights

### Use license

Open Data Commons Attribution License

## Data resources

### Data package title

Zoological Museum of the University of Athens

### Resource link

http://lifewww-00.her.hcmr.gr:8080/gbifgreece/resource.do?r=list_of_aves_of_the_zoological_museum_of_the_university_of_athens_

### Alternative identifiers

http://lifewww-00.her.hcmr.gr:8080/gbifgreece/resource.do?r=list_of_aves_of_the_zoological_museum_of_the_university_of_athens_

### Number of data sets

1

### Data set 1.

#### Data set name

Avian Collection – Zoological Museum of the University of Athens

#### Data format

Darwin Core Archive

#### Number of columns

34

#### Character set

UTF-8

#### 

**Data set 1.** 

Column label	Column description
identifiedBy	A list (concatenated and separated) of names of people, groups, or organizations who assigned the Taxon to the subject.
sex	The sex of the biological individual(s) represented in the Occurrence. This information is not always available. Specimens may not appear sexual dimorphism or sexual characteristics are indistinct.
decimalLatitude	The geographic latitude (in decimal degrees, using the spatial reference system given in geodeticDatum) of the geographic center of a Location. All locations were manualy georeferenced based on the information provided in the locality label. C﻿ountry borders and locality names ﻿at the time﻿ of the occurence were taken into account during the georeferencing.﻿
decimalLongitude	The geographic longitude (in decimal degrees, using the spatial reference system given in geodeticDatum) of the geographic center of a Location. All locations were manualy georeferenced based on the information provided in the locality label.﻿ Country borders and locality names at the time of the occurence were taken into account during the georeferencing.﻿
coordinateUncertaintyInMeters	The horizontal distance (in meters) from the given decimalLatitude and decimalLongitude describing the smallest circle containing the whole of the Location.
locality	The specific description of the place. This information is not available for all specimens.
country	The name of the country in which the Location occurs. This information is not available for all specimens. In some cases the locality information is much broader than a country (e.g. Asia). Also, country borders and locality names during the date of the occurence may differ from the current state.
locationRemarks	Comments or notes about the Location. Elaboration of the information were taken into account during the georeferencing.
recordedBy	A list of names of people, groups, or organizations responsible for recording the original Occurrence.
EventDate	The date-time or interval during which an Event occurred.
TaxonRemarks	The taxon or name first given to the specimen.
scientificNameID	An identifier for the nomenclatural details of a scientific name.
ScientificName	The full scientific name. This information is not always available. Bad condition of the specimens may inhibit identification.
Genus	The full scientific name of the genus in which the taxon is classified.
SpecificEpithet	The name of the first or species epithet of the scientificName. This information is not available for all individuals. Bad condition of the specimens may hinder identification.
scientificNameAuthorship	The authorship information for the scientificName formatted according to the conventions of the applicable nomenclaturalCode.
Order	The full scientific name of the order in which the taxon is classified.
Family	The full scientific name of the family in which the taxon is classified.
Kingdom	The full scientific name of the kingdom in which the taxon is classified.
Phylum	The full scientific name of the phylum or division in which the taxon is classified.
Class	The full scientific name of the class in which the taxon is classified.
nomenclaturalCode	The nomenclatural code (or codes in the case of an ambiregnal name) under which the scientificName is constructed.
countryCode	The standard code for the country in which the Location occurs.
DatasetName	The name identifying the data set from which the record was derived.
fieldΝumber	An identifier given to the event in the field.
occurenceID	An identifier for the Occurrence (as opposed to a particular digital record of the occurrence).
catalogNumber	An identifier for the record within the data set or collection.
institutionCode	The acronym in use by the institution having custody of the object or information referred to in the record.
ownerInstitutionCode	The acronym in use by the institution having ownership of the object or information referred to in the record.
preparations	The preservation method for a specimen.
basisOfRecord	The specific nature of the data record.
individualcount	The number of individuals represented present at the time of the Occurrence.
collectioncode	The current state of a specimen with respect to the collection identified in collectionCode or collectionID.
lifeStage	The life stage of the individual at the time the Occurrence was recorded. This information is not always available. Bad condition of the specimens may hinder lifestage determination.

## Supplementary Material

Supplementary material 1 ZMUA_Aves Data type: Occurence
Brief description: The dataset provides the list of avian specimens in the collection of the Zoological Museum of the University of Athens (ZMUA) along with all the rervant information availabe.
File: oo_108605.xlsx Gabriella Papastefanou, Igor Shogolev

## Figures and Tables

**Table 1.** List of Vulnerable, Endangered and Critically Endangered species (IUCN Red List of Threatened Species) available in the avian collection of ZMUA

**Vulnerable**	**Endangered**	**Critically Endangered**
*Acrocephalus paludicola*	*Emberiza aureola*	*Alcedo euryzona*
*Anser erythropus*	*Neophron percnopterus*	*Didunculus strigirostris*
*Aquila heliaca*	*Phalacrocorax capensis*	*Gyps rueppelli*
*Aythya ferina*		*Strigops habroptila*
*Balearica pavonina*		*Trigonoceps occipitalis*
*Cacatua moluccensis*		
*Catreus wallichii*		
*Clangula hyemalis*		
*Coeligena prunellei*		
*Columba eversmanni*		
*Dendroica cerulea*		
*Euphagus carolinus*		
*Fratercula arctica*		
*Goura cristata*		
*Heliodoxa gularis*		
*Lophura erythrophthalma*		
*Melanitta fusca*		
*Neomorphus geoffroyi*		
*Otis tarda*		
*Platyrinchus leucoryphus*		
*Podiceps auritus*		
*Procnias nudicollis*		
*Psittacus erithacus*		
*Puffinus yelkouan*		
*Pycnonotus zeylanicus*		
*Pyrrhura cruentata*		
*Ramphastos vitellinus*		
*Streptopelia turtur*		
*Syrmaticus reevesii*		
*Thripophaga macroura*		
*Touit surdus*		
*Tragopan melanocephalus*		
*Turdus feae*		
*Tympanuchus cupido*		
